# Long-Term Results of a Digital Hypertension Self-Management Program: Retrospective Cohort Study

**DOI:** 10.2196/43489

**Published:** 2023-08-24

**Authors:** Justin Wu, Jenna Napoleone, Sarah Linke, Madison Noble, Michael Turken, Michael Rakotz, Kate Kirley, Jennie Folk Akers, Jessie Juusola, Carolyn Bradner Jasik

**Affiliations:** 1 Omada Health Inc San Francisco, CA United States; 2 Google Mountain View, CA United States; 3 American Medical Association Chicago, IL United States; 4 Anchor Outcomes, LLC San Francisco, CA United States

**Keywords:** hypertension, digital health program, home measurement, self-management, behavior change

## Abstract

**Background:**

Digital health programs that incorporate frequent blood pressure (BP) self-monitoring and support for behavior change offer a scalable solution for hypertension management.

**Objective:**

We examined the impact of a digital hypertension self-management and lifestyle change support program on BP over 12 months.

**Methods:**

Data were analyzed from a retrospective observational cohort of commercially insured members (n=1117) that started the Omada for Hypertension program between January 1, 2019, and September 30, 2021. Paired *t* tests and linear regression were used to measure the changes in systolic blood pressure (SBP) over 12 months overall and by SBP control status at baseline (≥130 mm Hg vs <130 mm Hg).

**Results:**

Members were on average 50.9 years old, 50.8% (n=567) of them were female, 60.5% (n=675) of them were White, and 70.5% (n=788) of them had uncontrolled SBP at baseline (≥130 mm Hg). At 12 months, all members (including members with controlled and uncontrolled BP at baseline) and those with uncontrolled SBP at baseline experienced significant mean reductions in SBP (mean –4.8 mm Hg, 95% CI –5.6 to –4.0; –8.1 mm Hg, 95% CI –9.0 to –7.1, respectively; both *P*<.001). Members with uncontrolled SBP at baseline also had significant reductions in diastolic blood pressure (–4.7 mm Hg; 95% CI –5.3 to –4.1), weight (–6.5 lbs, 95% CI –7.7 to –5.3; 2.7% weight loss), and BMI (–1.1 kg/m^2^; 95% CI –1.3 to –0.9; all *P*<.001). Those with controlled SBP at baseline maintained within BP goal range. Additionally, 48% (418/860) of members with uncontrolled BP at baseline experienced enough change in BP to improve their BP category.

**Conclusions:**

This study provides real-world evidence that a comprehensive digital health program involving hypertension education, at-home BP monitoring, and behavior change coaching support was effective for self-managing hypertension over 12 months.

## Introduction

Hypertension, which is defined as systolic blood pressure (SBP) ≥130 mm Hg or diastolic blood pressure (DBP) ≥80 mm Hg, impacts nearly 1 in 2 adults in the United States and increases the risk for heart disease and stroke, 2 of the leading causes of death both in the United States and globally [1,2]. While hypertension is one of the most common primary care diagnoses [3], the Centers for Disease Control and Prevention estimates that just 1 in 4 adults (24%) with hypertension have their condition under control (defined as SBP <130 mm Hg and DBP <80 mm Hg) [4]. A strong relationship exists between rising SBP and DBP and increased risk of cardiovascular disease, including a 2-fold increased risk of death from stroke or heart disease for every 20 mm Hg increase in SBP [5].

Various interventions that incorporate evidence-based behavioral strategies such as self-monitoring of blood pressure (BP) [6-9] and healthy lifestyle changes, such as dietary modifications and increased physical activity [10-13] in conjunction with pharmacological treatments [1], have demonstrated effectiveness in the treatment of hypertension. To improve BP control at scale, translatable innovative solutions are needed that provide support for hypertension management both during traditional in-person office visits as well as at home, where individuals spend the majority of their time [14-19].

In support of using home blood pressure as part of the management of hypertension, the American Heart Association (AHA) and the American Medical Association reviewed the evidence for the use of home blood pressure monitoring (HBPM) [6]. HBPM is a stronger predictor of cardiovascular risk than office-based BP measurements [6,7]. In order to demonstrate clinically meaningful BP reductions that reduce the risk of developing a cardiovascular event [20,21], HBPM typically requires the use of education, human-led coaching interventions, and intensive clinical support [8,9].

Digital health and mobile health solutions combine the advantages of HBPM and the provision of data-driven insights with programs designed to support patients through self-management, goal setting, behavior change, and healthy lifestyle education [22]. Some digital solutions automate this support through artificial intelligence or other conversational technology (eg, texting) and self-service functions; however, automation has struggled with replicating the elements of building personal connection and promoting engagement found in human-led coaching programs [23-26].

As health care grapples with hypertension as a persistently undertreated and costly chronic disease, more pragmatic solutions are needed to address the significant time required to support lifestyle modifications, improve HBPM adherence, and respond to the real-time data being collected through HBPM to ensure that appropriate and timely care is being delivered.

The Omada for Hypertension program is a digital health behavior change solution that combines HBPM with human-led coaching support and a comprehensive lifestyle modification curriculum. A recent pilot study demonstrated that Omada for Hypertension members improved BP control after 3 months [27]; however, longer-term results of the program have yet to be investigated. The primary objective of this analysis is to examine the change in SBP between baseline and 12 months among Omada for Hypertension members. The secondary objectives are to (1) measure SBP change at 12 months compared to baseline among members whose SBP was uncontrolled at baseline (≥130 mm Hg [uncontrolled] and among members whose SBP was controlled at baseline <130 [controlled]); (2) examine weight change at 12 months overall and by SBP control status at baseline; and (3) assess change in DBP at 12 months compared to baseline overall and among those with uncontrolled BP at baseline.

## Methods

### Study Design

This was a nonrandomized, retrospective observational cohort study evaluating the clinical outcomes from baseline to 12 months among commercial health plan members enrolled in the Omada for Hypertension program. To enroll in the Omada for Hypertension program, individuals were required to meet the following criteria: (1) have coverage from their employer or health insurance plan for the benefit; (2) be ≥18 years old; (3) have a self-reported diagnosis of hypertension; and (4) not have any medical contraindications. All members self-enrolled in the program and were not compensated for their participation.

To be included in the current analysis, members met the following criteria: (1) previously or currently enrolled in the Omada for Hypertension program; (2) completion of baseline and 12-month home BP readings (following the procedures described in the Measures section below); and (3) a program start date (defined as the date of first home BP reading uploaded) between January 1, 2019, and September 30, 2021.

### Ethical Considerations

The study was a secondary analysis of previously collected deidentified commercial data and thus was deemed exempt from ethics approval by the WCG institutional review board (confirmation ID: 45104379).

### Program Description

The Omada for Hypertension program is a digitally delivered hypertension self-management program that pairs asynchronous human support through health coaches and hypertension education specialists with a virtual platform that is accessed either through a website or through an app available on web-enabled devices (eg, smartphone and tablet). The program offers both a hypertension education curriculum and comprehensive lifestyle self-management support using behavior change techniques, in addition to a cellularly connected BP cuff for HBPM (BodyTrace, Inc) and a cellularly connected digital scale (Greater Goods, LLC or BodyTrace, Inc).

Program members are paired with an Omada care team, which includes a health coach and a hypertension education specialist (ie, Certified Diabetes Care and Education Specialist trained for hypertension) who communicate with members through an asynchronous messaging platform. Members are instructed to take BP measurements at home on a monthly basis (per the protocol described in the Measures section below), as well as prior to a scheduled visit with their doctor, after a change in medication, and according to their doctor’s instructions. The health coach supports members’ progress throughout the program, provides feedback to members regarding HBPM, encourages medication adherence, and prepares members for their doctor visits. The hypertension education specialist reviews members’ BP data and provides individual feedback and counseling on nonpharmacological treatment for BP pattern management as needed.

Additional program components include hypertension education content, lifestyle modification advice (eg, physical activity and individualized dietary support), social support, goal-setting tools, self-monitoring capabilities via cellularly connected devices, feedback on self-monitoring data, BP pattern management, and check-ins with members to encourage communication with their health care providers when adjustments to medication or care may be needed. Recommendations made by the Omada care team are done in alignment with the member’s care plan created by their regular treating provider. In addition, members are placed in a virtual peer support group and can communicate with other program members through a secure group discussion board.

### Measures

#### Blood Pressure

Members measure their BP (mm Hg) with a Food and Drug Administration-cleared cellularly connected BP device, which is connected to the member’s account and requires no additional setup. Members also had the option of using their own BP device and manually reporting their readings in the app or website, but only approximately 2% of members used this option. Members were provided with instructions for how to perform accurate BP measurements at home [28] and were instructed to establish a baseline BP reading by choosing a 3-day window where they measured their BP twice in the morning and twice in the evening for each of the 3 consecutive days. Additionally, members were encouraged to take monthly BP readings using the same protocol as above as part of the self-monitoring hypertension program.

Members’ BP measurements were averaged if the measurements met the following minimum criteria at baseline and 12-month follow-up: (1) 3 or more measurements taken over at least 3 days with at least ≥1 measurement collected per day or (2) ≥4 or more measurements taken over at least 2 days with ≥2 measurements collected per day [29]. The baseline and 12-month BP data were calculated as the average of the BP measurements meeting the minimum criteria closest to the program start date and up to 30 days after the program start date and closest to 12 months and up to 15 months from the program start date, respectively. At baseline, uncontrolled SBP was defined as ≥130 mm Hg (controlled <130 mm Hg), and uncontrolled DBP was defined as ≥80 mm Hg (controlled <80 mm Hg) [30]. BP measurements were used to categorize members into HBPM categories that correspond with current AHA/American College of Cardiology 2017 recommendations and are appropriate thresholds for HBPM [30].

To keep consistent with AHA/American College of Cardiology–recommended treatment goals for those with hypertension, the normal and elevated HBPM categories were consolidated into an “At Goal” category. Thus, HBPM categories were defined as (1) At Goal: SBP<130 mm Hg and DBP<80 mm Hg, (2) Stage 1: SBP between 130 and 134 mm Hg or DBP between 80 and 84 mm Hg, and (3) Stage 2: SBP ≥135 mm Hg or DBP≥85 mm Hg [30]. If a member’s BP falls into 2 different categories, the member is assigned to the higher HBPM category. Consistent with recent meta-analyses, a clinically meaningful change in SBP over the study period was defined as a 5 mm Hg reduction [20,21].

#### Body Weight

Body weight (lbs) was collected with an Omada-provided cellularly connected digital scale, which is linked to the member’s account. The baseline and 12-month data were calculated as the average of all weight measurements on the day with at least 1 weight measurement closest to the program start date within the window of 7 days prior to and 30 days post the program start date and within an 11- to 13-month window from the program start date, respectively. Absolute change in body weight and percent weight loss from baseline to 12 months was calculated. BMI was calculated from self-reported height and measured weight and categorized into an obesity indicator variable (obesity ≥30 kg/m^2^ vs without obesity <30 kg/m^2^).

#### Member Characteristics

Members self-reported their demographic information, including sex (male and female), race and ethnicity (White, Black, Hispanic, Asian, and other), annual income (>US $50,000 and ≤US $50,000), and educational attainment (college education and above and less than college education), upon enrollment in the program. Self-reported medication usage was collected during account setup and further categorized into a 2-level variable for whether members were taking hypertension-related medications versus not at baseline [31].

#### Program Engagement

Program engagement was measured by counts of actions taken within the program app or website each week in the program and included six components: (1) number of conversations started on a group discussion board; (2) number of comments (replies to conversations) made on the group discussion board; (3) number of hearts (likes) shared on the group discussion board; (4) number of messages sent to their Omada care team; (5) number of meals tracked; and (6) number of lessons completed. The median of the average weekly activities for all members was used as the cutoff point for high versus low engagement (ie, high engagement ≥4 activities per week, normal or low engagement <4 activities per week).

Additionally, activities related to self-monitoring behaviors were captured by the average number of times per week that members recorded a physical activity as well as the average number of times per week that members recorded weight. BP engagement was assessed by the average times per month that members used their BP cuff or manually entered BP readings.

### Statistical Analysis

Differences in baseline demographic characteristics and clinical measures between members with uncontrolled (≥130 mm Hg) versus controlled (<130 mm Hg) SBP at baseline were tested using chi-square and *t* tests. Changes in clinical outcomes (SBP, DBP, weight, and BMI) from baseline to 12 months were assessed using paired *t* tests. Differences in the proportion of hypertension members within each stage (At Goal, Stage 1, and Stage 2) were analyzed using a marginal homogeneity test of symmetry to determine whether there was a significant shift of members within each stage from baseline to 12 months. Linear regression was used to model the change in SBP at 12 months overall and stratified by SBP control status at baseline. Unadjusted, minimally adjusted (age, sex, and race and ethnicity), and fully adjusted models (minimally adjusted covariates, program engagement, and obesity status) were fit. Model assumptions and goodness of fit tests were assessed, and the fully adjusted model was selected as the final model. All analyses used 2-sided hypothesis testing and were conducted in R (version 4.1; R Foundation) and Stata (version 17.0; StataCorp).

## Results

### Sample Characteristics

**Figure 1:**
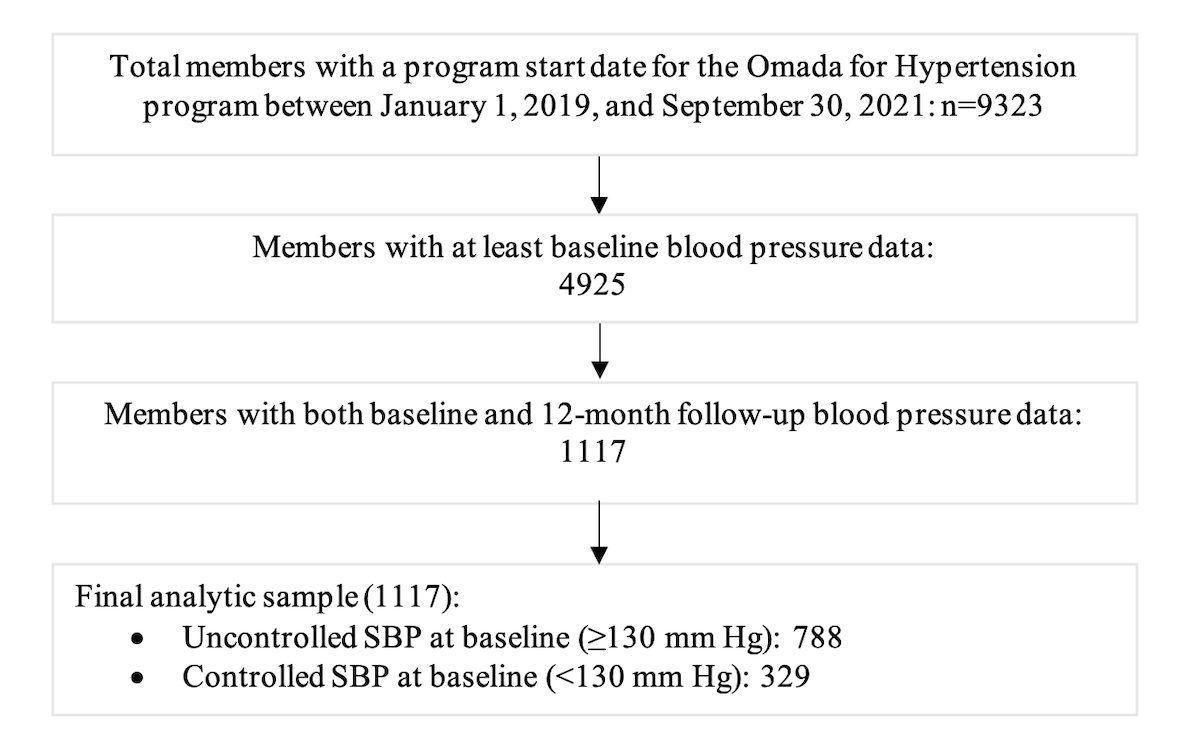
Omada for Hypertension member enrollment and study participation flow chart. SBP: systolic blood pressure.

The final sample size for Omada for Hypertension members with baseline and 12-month BP data over the analysis period was 1117 members, including 788 members with uncontrolled SBP (≥130 mm Hg) at baseline and 329 with controlled SBP (<130 mm Hg) at baseline (Figure 1). The mean age of the overall sample was 50.9 (SD 9.6) years old, with approximately half of the overall sample self-identifying as female, 60.5% (n=675) of them self-identifying as White, and the majority reporting incomes over US $50,000 with at least a college education (Table 1).

Approximately two-thirds (n=724, 66.9%) of members were classified as obese, and 77% (n=860) of them had Stage 1 or 2 hypertension based on baseline SBP or DBP measurements. Stratified by SBP control at baseline, those with uncontrolled SBP were significantly more likely to be male and had significantly higher mean weight, BMI, and DBP at baseline (all *P*<.05; Table 1).

**Table 1:** Demographic characteristics, weight, and blood pressure overall and by systolic blood pressure (SBP) control status at baseline (uncontrolled SBP ≥130 mm Hg vs controlled <130 mm Hg).^a^

						Overall (n=1117)			SBP^b^ ≥130 (n=788; 70.5%)			SBP <130 (n=329; 29.5%)			*P* value	
**Demographic characteristics**																
	Age (years), mean (SD)				50.9 (9.6)			51.2 (9.5)			50.3 (10.0)			.13		
	**Age distribution (years), n (%)**														.63	
		18-44		284 (25.4)			194 (24.6)			90 (27.4)						
		45-64		768 (68.8)			548 (69.5)			220 (66.9)						
		≥65		65 (5.8)			46 (5.8)			19 (5.8)						
	**Sex, n (%)**														*.02*	
		Female		567 (50.8)			382 (48.5)			185 (56.2)						
		Male		550 (49.2)			406 (51.5)			144 (43.8)						
	**Income, n (%)**														.28	
		>US $50,000		600 (75.3)			435 (76.3)			165 (72.7)						
		≤US $50,000		197 (24.7)			135 (23.7)			62 (27.3)						
	**Race and ethnicity, n (%)**														.06	
		White		675 (60.5)			474 (60.5)			201 (61.3)						
		Black		187 (16.8)			147 (18.7)			40 (12.2)						
		Hispanic		114 (10.2)			76 (9.7)			38 (11.6)						
		Asian		99 (8.9)			64 (8.1)			35 (10.7)						
		Other		40 (3.6)			26 (3.3)			14 (4.3)						
	**Education, n (%)**														.18	
		College education and above		645 (66.3)			451 (65.0)			194 (69.5)						
		Less than a college education		328 (33.7)			243 (35.0)			85 (30.5)						
**Clinical measures**																
		Weight (lbs), mean (SD)		219.9 (51.1)			226.4 (52.0)			204.6 (45.2)			*<.001*			
		BMI (kg/m^2^), mean (SD)		35.2 (9.4)			36.3 (9.7)			32.7 (8.1)			*<.001*			
		**Obesity status (kg/m^2^), n (%)**													*<.001*	
			BMI≥30 kg/m^2^	724 (66.9)			544 (71.2)			180 (56.6)						
			BMI<30 kg/m^2^	358 (33.1)			220 (28.8)			138 (43.4)						
		**Hypertension medications, n (%)**													.69	
			Yes	620 (86.1)			434 (85.8)			186 (86.9)						
			No	100 (13.9)			72 (14.2)			28 (13.1)						
		SBP (mm Hg), mean (SD)		136.5 (13.4)			142.6 (10.8)			122.0 (5.6)			*<.001*			
		DBP^c^ (mm Hg), mean (SD)		82.4 (9.1)			85.4 (8.6)			75.1 (5.3)			*<.001*			
		**DBP control (mm Hg**), **n (%)**													*<.001*	
			≥80 mm Hg (uncontrolled)	676 (60.5)			604 (76.7)			72 (21.9)						
			<80 mm Hg (controlled)	441 (39.5)			184 (23.4)			257 (78.1)						
		**HBPM^d^ hypertension categories (based on baseline SBP and DBP), n (%)**													*<.001*	
			At Goal	257 (23.0)			0			257 (78.1)						
			Stage 1	383 (34.3)			323 (41.0)			60 (18.2)						
			Stage 2	477 (42.7)			465 (59.0)			12 (3.7)						

^a^Italicized *P* values are significant.

^b^SBP: systolic blood pressure

^c^DBP: diastolic blood pressure.

^d^HBPM: home blood pressure monitoring.

### Program Engagement

Overall, 55.5% (n=620) of members were classified as “highly engaged” with the program (mean weekly activities ≥4) versus 44.5% (n=497) as “less engaged” (weekly activities <4). Meal tracking accounted for 82% of the mean weekly engagement metric. There was no significant difference in program engagement by uncontrolled versus controlled SBP at baseline (*P*=.08). With regard to BP engagement, members used their BP cuff or manually entered BP values an average of 13.4 (median 8.7) times per month over 12 months. For self-monitoring related app or website activities, members weighed in on average 4 times per week and tracked their physical activity on average 3.7 times per week.

### Clinical Outcomes

As shown in Table 2, overall members had significant unadjusted mean reductions in SBP and DBP from baseline to 12 months (mean –4.8, 95% CI –5.6 to –4; mean –3.0, 95% CI –3.5 to –2.5, both *P*<.001, respectively). In addition, members with uncontrolled SBP at baseline experienced clinically meaningful reductions in SBP and DBP from baseline to 12 months (mean –8.1, 95% CI –9.0 to –7.1; mean –4.7, 95% CI –5.3 to –4.1, both *P*<.001, respectively). Nearly all members (604/676, 89%) with uncontrolled DBP (≥80 mm Hg) at baseline also had uncontrolled SBP at baseline. Members with uncontrolled DBP experienced clinically meaningful unadjusted mean reductions in DBP from baseline to 12 months (n=676; mean –5.7; *P*<.001). Members with controlled SBP at baseline experienced mean increases in SBP and DBP from baseline to 12 months (n=329; mean 3.1, 95% CI 2.0-4.2; 1.0, 95% CI 0.2-1.8, both *P*<.05, respectively), but these increases were not clinically significant (Table 2).

**Table 2:** Mean (95% CI) change over time in clinical outcomes from baseline to 12 months overall and by systolic blood pressure (SBP) control status at baseline (≥130 mm Hg vs <130 mm Hg; n=1117).^a^

Variable		Baseline, mean (95% CI)	12 months, mean (95% CI)	12-month change, mean (95% CI)	*P* value
**Overall**					
	SBP (mm Hg; n=1117)	136.5 (135.8 to 137.3)	131.8 (131.0 to 132.5)	–4.8 (–5.6 to –4.0)	<.001
	DBP^b^ (mm Hg; n=1117)	82.4 (81.9 to 83.0)	79.4 (78.9 to 79.9)	–3.0 (–3.5 to –2.5)	<.001
	Weight (lbs; n=973)	220.5 (217.4 to 223.7)	214.3 (211.2 to 217.4)	–6.2 (–7.2 to –5.3)	<.001
	BMI (kg/m^2^; n=966)	35.3 (34.8 to 35.9)	34.3 (33.8 to 34.9)	–1.0 (–1.2 to –0.9)	<.001
**Uncontrolled SBP ≥130 mm Hg at baseline**					
	SBP (mm Hg; n=788)	142.6 (141.9 to 143.4)	134.6 (133.7 to 135.4)	–8.1 (–9.0 to –7.1)	<.001
	DBP (mm Hg; n=788)	85.5 (84.9 to 86.1)	80.8 (80.1 to 81.4)	–4.7 (–5.3 to –4.1)	<.001
	Weight (lbs; n=685)	226.8 (222.9 to 230.6)	220.3 (216.5 to 224.1)	–6.5 (–7.7 to –5.3)	<.001
	BMI (kg/m^2^; n=681)	36.3 (35.6 to 37.1)	35.3 (34.6 to 36.0)	–1.1 (–1.3 to –0.9)	<.001
**Controlled SBP <130 mm Hg at baseline**					
	SBP (mm Hg; n=329)	122.0 (121.4 to 122.6)	125.1 (124.0 to 126.2)	+3.1 (+2.0 to +4.2)	<.001
	DBP (mm Hg; n=329)	75.1 (74.6 to 75.7)	76.2 (75.3 to 77.0)	+1.0 (+0.2 to +1.8)	.01
	Weight (lbs; n=288)	205.7 (200.6 to 210.8)	200.1 (195.3 to 204.9)	–5.6 (–7.3 to –3.9)	<.001
	BMI (kg/m^2^; n=285)	33.0 (32.1 to 33.9)	32.1 (31.2 to 33.0)	–0.9 (–1.2 to –0.6)	<.001

^a^12-month change=12 months–baseline; negative 12-month change indicates improvement in clinical outcomes.

^b^DBP: diastolic blood pressure.

The final multivariable regression models were consistent with unadjusted estimates (data not shown). Overall, in fully adjusted models, average marginal estimates of SBP significantly decreased by 4.8 mm Hg from baseline to 12 months (95% CI –5.6 to –4.0; *P*<.001), and among those with uncontrolled SBP at baseline, average marginal estimates of SBP significantly decreased by 8.0 mm Hg at 12 months (95% CI –9.0 to –7.1; *P*<.001). Among members with uncontrolled SBP at baseline, program engagement was significantly related to a 12-month change in SBP with highly engaged members experiencing greater mean reductions than less engaged members (–4.4 mm Hg; 95% CI –6.4 to –2.5; *P*<.001). Additionally, members with obesity had significantly greater reductions in SBP compared to those who did not have obesity (*P*=.02).

**Figure 2:**
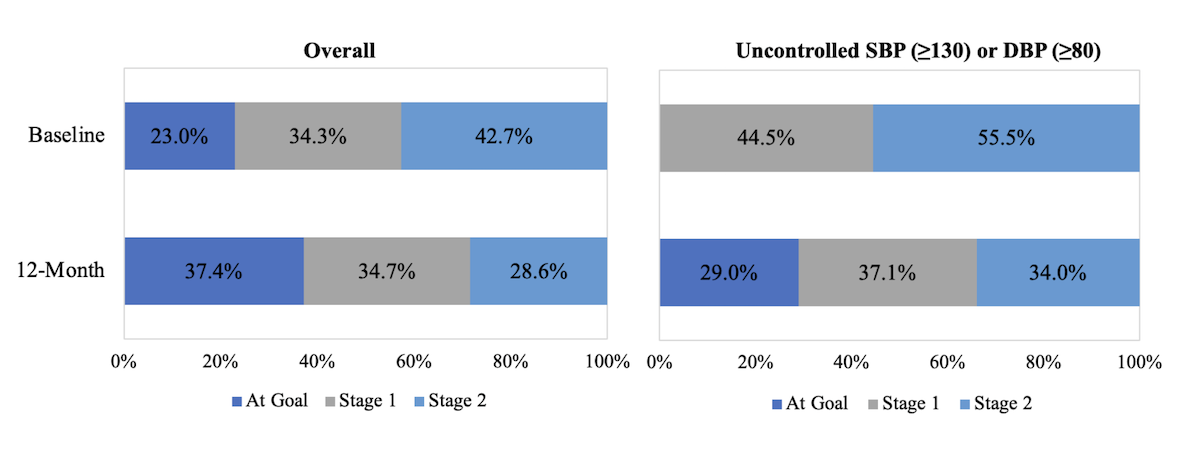
Home blood pressure monitoring hypertension category percentages at baseline and 12 months overall (n=1117) and among those with uncontrolled SBP (≥130 mm Hg) or DBP (≥80 mm Hg) at baseline (n=860). The figure on the left represents all members in the study (n=1117) and the figure on the right includes only those with uncontrolled SBP or DBP at baseline (n=860); *P* value <.001 overall and by SBP control status at baseline using marginal homogeneity test for shift in ordered proportion of members within home blood pressure monitoring hypertension stages at 12 months compared to baseline; home blood pressure monitoring categories were defined as: (1) At Goal: SBP<130 mm Hg and DBP<80 mm Hg, (2) Stage 1: SBP between 130 and 134 mm Hg or DBP between 80 and 84 mm Hg, and (3) Stage 2: SBP ≥135 mm Hg or DBP≥85 mm Hg. DBP: diastolic blood pressure; SBP: systolic blood pressure.

Both overall and among those with uncontrolled SBP or DBP at baseline, the percentage of members within each HBPM hypertension category (ie, At Goal, Stage 1, and Stage 2) showed significant improvement from baseline to 12 months as shown in Figure 2 (*P*<.001). Overall, 13% (146/1117) of members shifted from Stage 1 to At Goal, 9% (103/1117) from Stage 2 to At Goal, and 15% (169/1117) from Stage 2 to Stage 1. Additionally, among those with uncontrolled BP at baseline, 17% (146/860) of members shifted from Stage 1 to At Goal, 12% (103/860) from Stage 2 to At Goal, and 19.7% (169/860) from Stage 2 to Stage 1.

**Figure 3:**
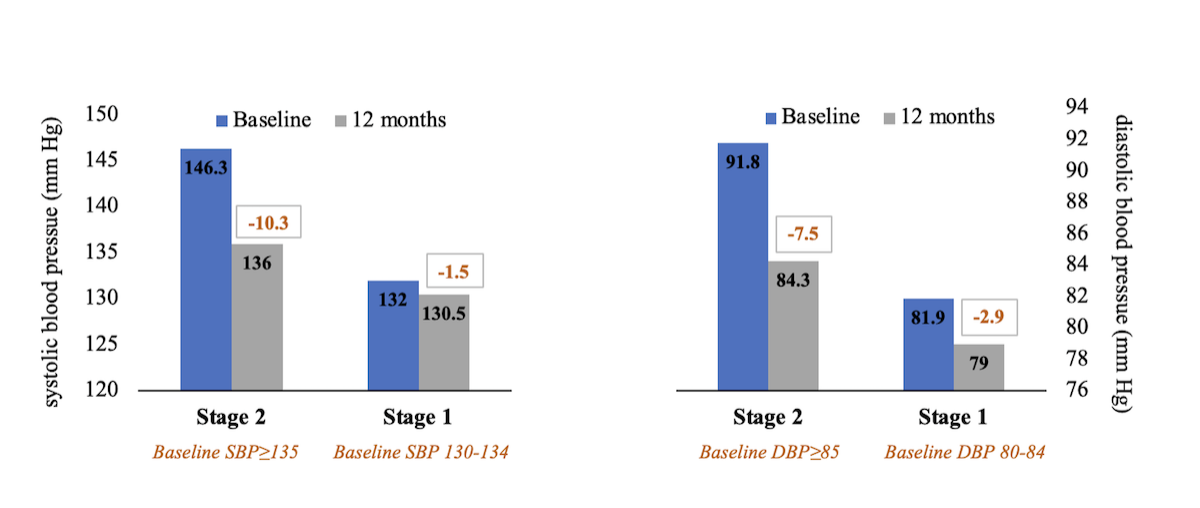
Mean 12-month reduction in SBP and DBP by baseline hypertension categories among those with uncontrolled SBP (≥130 mm Hg) or uncontrolled DBP (≥80 mm Hg) at baseline (n=860). Within category change *P* values all <.001. DBP: diastolic blood pressure; SBP: systolic blood pressure.

The mean change in BP by HBPM stage among those with uncontrolled SBP and DBP at baseline is shown in Figure 3. Members with Stage 2 SBP and DBP at baseline demonstrated clinically meaningful and statistically significant reductions in BP at 12 months (–10.3 and –7.5 mm Hg, respectively; both *P*<.001); furthermore, the mean DBP at 12 months moved to the At Goal category for those with Stage 1 DBP at baseline (Figure 3).

Overall, members lost on average 6.2 lbs at 12 months (95% CI –7.2 to –5.3; *P*<.001) and approximately 2.6% weight loss. Both members with uncontrolled and controlled SBP at baseline experienced significant weight loss from baseline to 12 months (–6.5 lbs, 95% CI –7.7 to –5.3; –5.6 lbs, 95% CI –7.3 to –3.9, respectively; both *P*<.001); the mean percent weight loss among those with uncontrolled versus controlled SBP at baseline were 2.7% and 2.4% at 12 months, respectively (Table 2).

## Discussion

### Principal Findings

Members with uncontrolled BP at baseline experienced significant and clinically meaningful mean reductions in SBP and DBP from baseline to 12 months. When examining clinical improvement through the lens of shifting hypertension categories, more than 48% (418/860) of members with uncontrolled SBP or uncontrolled DBP at baseline experienced a large enough improvement in BP to shift down by 1 or more categories. Weight and BMI addressed in the program through diet, physical activity, and behavior change support also showed statistically significant reductions during the study period. Program engagement was also evident in this study, with over half of members being classified as “highly engaged” (mean weekly activities ≥4). These members experienced significantly greater reductions in SBP compared to those that were less engaged in the program, supporting current literature on the effectiveness of engagement on interventions for hypertension self-management [32,33].

Although members with SBP and DBP within the goal range at baseline experienced statistically significant increases in BP at 12 months, the magnitude of the changes was not clinically meaningful, and the mean of this group still fell within the At Goal HBPM category at 12 months. These increases may be due to regression to the mean, random variability in the data, or from disease progression as members’ age in the program. Moreover, these members also lost nearly as much percent body weight as members with uncontrolled BP at baseline, demonstrating that they also benefited from the program from a weight loss perspective.

While there have been a number of studies examining different models of digital health solutions for the management of hypertension [22,34], few have investigated real-world outcomes in programs that incorporate the three specific components included in the Omada for Hypertension program: (1) self-monitoring of BP through HBPM, (2) comprehensive lifestyle education and behavior change interventions, and (3) human-led health coaching to support behavior change [35-38]. The spectrum of digital health interventions may also include the involvement of licensed clinicians as part of the care team (eg, pharmacists and physicians), automated education or direct medication reminders via text, electronic pill box or medication bottles, physical activity trackers, and other wearable devices.

In a review of the literature, 1 previous cohort study of a hypertension self-management program offered through an employer health plan also found that higher engagement with the app was associated with greater reductions in SBP [38]. The program intervention in that study and the Omada for Hypertension program are similar in that they both offer hypertension self-management with a BP monitor and connected smartphone app; however, a difference is that the former uses an automated lifestyle coaching platform, whereas the Omada program uses human-led behavior change coaching. Likewise, in the HOME BP trial [39], which examined a digital intervention for hypertension with self-monitoring of BP and guided self-management, SBP control status was improved after 1 year compared to usual care. In the HOME BP intervention, however, all members received a BP medication review by a clinician with an individualized drug titration plan, directly addressing the issue of therapeutic inertia. These examples show that the Omada for Hypertension program outcomes are consistent with other studies; however, direct comparisons in the literature are difficult to make due to the variety of intervention designs.

### Limitations

There are limitations to this study. First, the study population was composed primarily of commercially insured and middle-aged adults, with a lower representation than the national average of members who identify as Hispanic. All members had access to internet connectivity, a smartphone app, a web OS platform, a computer or tablet, and sufficient technology literacy. These demographics limit the generalizability of our findings to other populations including Medicaid, Medicare, or uninsured populations, which currently have lower adoption of technology.

Second, members were enrolled in the program based on a self-reported diagnosis of high BP or hypertension, and access to medical records was not available to further validate the diagnosis. An assessment of usual medical care and the presence of medication adherence issues and treatment inertia was also not possible due to the lack of longitudinal medication data. In addition, we accounted for normal variation in BP by calculating a mean BP using clinical best practices rather than relying upon a single BP measurement [29]; yet, the potential for measurement error may remain. Moreover, as a nonrandomized, observational cohort study, the lack of a control group may introduce confounding variables and bias, and findings could be attributable in part to regression to the mean.

Due to the real-world digital delivery of this program, about one-quarter of members with a baseline BP measurement also had a valid 12-month BP follow-up measurement. However, there were only a few differences between those with complete 12 months of data versus those without or excluded from the study; excluded members due to missing follow-up data were more likely to be Black and female (both *P*<.001). Additionally, some members may have been engaged with the program even if they did not report BP data or chose to only report limited BP data at 12 months, thus not meeting the minimum criteria to be included in the study (per the protocol discussed in the measures section of the methods).

Lastly, the mean weekly engagement metric included a wide range of activities that do not carry equal weight in terms of the amount of activation required and the evidence supporting their effectiveness. Nevertheless, we summed all 6 measures of action-oriented engagement to create a total mean weekly engagement metric for a number of reasons: members may uniquely benefit from different types of content and interactions to help promote behavior change and clinical improvements and may have more opportunities to engage in some engagement metrics than others (ie, can log 3+ meals per day but only have the opportunity to complete 1 lesson per week).

### Future Directions

Future research should examine the implementation of similar digital health programs into the health care ecosystem including bidirectional data exchange and care coordination. More evidence for the applicability of digital programs to other populations (eg, Medicare and Medicaid) and how a digital solution might reduce the burden on the primary care team would further bolster the evidence for a widespread adoption of this approach to hypertension management.

The cost-effectiveness and the long-term impact of a digital health program on the health economics of hypertension management were not directly assessed in this study. However, a recent study showed that patients with hypertension had a US $432 decrease in health care expenditures in the year following a decrease in 1 BMI unit inflated to 2022 dollars [40]. Thus, further analysis of the economic impact and potential savings of digital hypertension self-management programs is warranted.

### Conclusions

The current management of hypertension in the United States is in need of innovative solutions that can scale to reverse the alarming trend of worsening disease and unsustainable costs related to hypertension in the health care system. Digital health solutions have shown promise in allowing more care to be shared by the patient in a self-management program and less time required by the physician to deliver lifestyle counseling and behavior change support. This study provides evidence that a digital solution, coupled with human-led health coaching, is associated with improved SBP control over a sustained period of time.

## Abbreviations

AHA: American Heart Association
BP: blood pressure
DBP: diastolic blood pressure
HBPM: home blood pressure monitoring
SBP: systolic blood pressure
